# Hypertrophic Pyloric Stenosis Following Repair of Esophageal Atresia and Tracheo-Esophageal Fistula

**Published:** 2014-07-10

**Authors:** Emmanuelle Seguier-Lipszyc, Baruch Klin

**Affiliations:** Pediatric Surgery Department, Assaf Harofeh Medical Center, Zerifin 70300, Israel

**Keywords:** Tracheo-Esophageal fistula, Esophageal atresia, Hypertrophic pyloric stenosis

## Abstract

Two cases of hypertrophic pyloric stenosis (HPS) developed after a few weeks of repair of an esophageal atresia and tracheo-esophageal fistula (EA and TEF). Both cases were dealt successfully with laparoscopic pyloromyotomy.

## INTRODUCTION

HPS following esophageal atresia repair is rarely reported. Regurgitation and vomiting in operated cases of EA and TEF is attributed mainly to gastroesophageal reflux (GER) or anastomotic stricture [1-3]. Early diagnosis and treatment is mandatory for good prognosis which may be a dilemma. We present two cases of HPS following an EA and TEF repair successfully treated laparoscopically. 

## CASE SERIES

**Case 1:** A 2865-g boy was born at 36-wk gestational age in our institution. Because of respiratory distress, a chest x-ray was performed, showing the nasogastric tube rolled at the distended upper esophageal pouch. A right thoracotomy, ligation of the fistula and primary anastomosis were performed on his 3rd day of life. Postoperative contrast study of the esophagus and stomach a week later was normal. A VACTERL association was present with bilateral hydronephrosis (known from antenatal studies), and atrial and ventricular septal defects. The post-operative course was uneventful and the baby was discharged on the 15th postoperative day. At 5 weeks of age, vomiting after every feed developed. An abdominal ultrasound was performed showing a hypertrophic pylorus. Following intravenous fluid therapy for a day, a laparoscopic pyloromyotomy was uneventfully performed and the child was discharged the next day on full feeding regimen.

**Case 2:**A 3335-g girl was born at 40-wk gestational age in our institution and diagnosed as EA and TEF due to hypersalivation and intolerance to feeding. A right thoracotomy, ligation of the fistula, and primary anastomosis were performed on her 2nd day of life. Postoperative contrast study of the esophagus and stomach a week later was normal. She also had an anteriorly placed anus which did not require surgical treatment at that time. The post-operative course was uneventful and the baby was discharged on the 17th postoperative day. At 7.5 weeks of life, she again presented with recurrent vomiting for 3 days. An abdominal ultrasound was performed showing a hypertrophic pylorus. A laparoscopic pyloromyotomy was uneventfully performed and the child discharged the next day on full feeding regimen. The mother also had a pyloromyotomy in her infancy.

## DISCUSSION

The incidence of additional congenital anomalies in patients with EA and TEF is approximately 50% [1,2]. In a review of all HPS in the state of New York during 7 years, out of all the children with HPS 7% had a major malformation compared with 3.7% of the general population. Three major malformations occurred more frequently in children with HPS: intestinal malrotation, obstructive defects of the urinary tract, and esophageal atresia [3].

HPS complicating the postoperative period of EA and TEF repair is rarely reported in literature [2,4-7]. The association of EA and TEF with HPS was first reported in 1969 [8]. A series of 10 newborns who developed HPS after abdomino-thoracic surgical procedure was reported by Nasr and Ein in 2007 [9]. A recent publication showed an unexplained high incidence of 7.5% of HPS after EA repair [10]. Postoperative vomiting in neonates who have had important abdomino-thoracic surgery in the early days of life is a common occurrence and especially after EA and TEF repair. The diagnosis of HPS was never suspected on clinical grounds in any of these babies and was made only by ultrasonography or by contrast studies. In our series, ultrasound also helped us in diagnosis of HPS although our initial differentials were GER and anastomotic stricture. In one, patient the mother also had HPS during her infancy led us to early diagnosis. Laparoscopy is well tolerated in neonates despite their high sensitivity to insufflations. The first laparoscopic extramucosal pyloromyotomy was described in 1991 [11,12]. We found no technical problem in laparoscopic pyloromyotomy in both of our patients.

To conclude, despite of its rarity, HPS as a cause of recurrent vomiting following EA and TEF repair should be kept in mind in order to avoid delay in diagnosis and treatment.

## Footnotes

**Source of Support:** Nil

**Conflict of Interest:** None

